# Epidemiology, species distribution, antifungal susceptibility, and outcome of candidemia in intensive care units in Isfahan, Iran

**DOI:** 10.18502/cmm.8.3.11217

**Published:** 2022-09

**Authors:** Azam Haghighatfard, Saeed Abbasi, Pegah Alijani, Farzaneh Afyooni Akbari, Hossein Rashidi, Parvin Dehghan

**Affiliations:** 1 Department of Parasitology and Mycology, School of Medicine, Isfahan University of Medical Sciences, Isfahan, Iran; 2 Head of Anesthesiology and Critical Care Research Center, Nosocomial Infection Research Center, Isfahan University of Medical Sciences, Isfahan, Iran; 3 Ayatollah Boroujerdi Hospital, Department of Microbiology, Lorestan University of Medical Sciences, Lorestan, Iran; 4 Department of Medical Parasitology and Mycology, School of Public Health, Tehran University of Medical Sciences, Tehran, Iran; 5 Ayatollah Boroujerdi Hospital, Head of laboratory, Lorestan University of Medical Sciences, Lorestan, Iran

**Keywords:** Candidemia, Identification, Intensive care unit, PCR, Yeast

## Abstract

**Background and Purpose:**

Candidemia is known as an invasive fungal infection with high mortality. The prevalence of candidemia in intensive care unit (ICU) patients is more than in other hospital wards. Early diagnosis of candidemia in these patients is essential for disease management.

**Materials and Methods:**

This study included 250 patients suspected of candidemia. Blood samples were taken from patients and incubated. The fungal isolates were identified by PCR-RFLP method using *MSP I* restriction enzyme. Demographic characteristics, risk factors, underlying diseases, and laboratory analysis results were mined in this study.

**Results:**

In total, 22 blood samples were identified as positive for *Candida* yeasts in culture. The most common underlying diseases in these patients were heart disease and hypertension (36.4%). *Candida albicans* with 12 cases (54.5%) was the most isolated species, followed by *C. parapsilosis* (n=5, 22.7%), *C. glabrata* (n=4, 18.2%), and *C. tropicalis* (n=1, 4.5%) in descending order. Intravenous catheter use was recognized as the most common risk factor in patients with candidemia (77.3%), and after that, the use of mechanical ventilation (68.2%) and urinary catheter (40.9%) obtained the highest frequency. Furthermore, 17 patients were prescribed at least one antifungal drug, of which fluconazole was the most used (36.4%). The mortality rate in patients in this study was 63.6%. All *C. albicans* isolates were susceptible to antifungal agents but in non-*albicans Candida* (NAC), drug resistance to fluconazole, voriconazole, and caspofungin were observed.

**Conclusion:**

Although *C. albicans* was the most common fungal species in this study, the prevalence of NAC species was high. The increasing frequency of NAC species is a concern because they have different patterns of drug resistance. Recognition of risk factors in patients admitted to ICUs can help prevent candidemia or properly manage the disease.

## Introduction

*Candida* is normally part of the skin microbial flora; however, in immunocompromised patients (e.g., those with diabetes), prolonged hospitalization in ICU, human immunode-ficiency virus, and malignancy can cause severe systemic infections [ 1 , 2 ]. In terms of morbidity and mortality, candidemia is a very important infection caused by several species of *Candida* and should be diagnosed and treated immediately [ 3 ]. Despite advances in disease management and diagnosis of candidemia, *Candida* species remain one of the main causes of mortality in yeast infections in patients with underlying conditions [ 4 , 5 ]. 

Based on the yeast species, the mortality rate is reported to be between 30% to 85% [ 6 ]. Although *C. albicans* has been reported as a common cause of candidemia in many studies, non-*albicans* species are also increasing [ 7 - 11 ]. The most important challenge in facing infections caused by non-*albicans Candida* (NAC) species is their resistance to conventional antifungal drugs [ 6 , 12 ]. The prevalence of candidemia in patients admitted to ICUs has been reported to be 10 to 20 times more than those in other hospital wards [ 13 ]. Bloodstream infections are the third site of infection in ICU patients. Using intravascular catheters is the most important predisposing factor for candidemia in these patients [ 14 ]. Different types of invasive candidiasis, including candidemia, present many challenges in ICUs. Diagnosis and treatment of these infections are usually time-consuming and followed by a positive blood culture; accordingly, the rate of mortality has increased. Early diagnosis and treatment of these infections can reduce the rate of mortality and high treatment costs [ 15 ]. The present study investigated ICU patients suspected of candidemia.

## Materials and Methods

This prospective study was conducted on patients admitted to the ICU from January 2018 to December 2020. A total of 250 patients suspected of fungemia were admitted to the ICU of Al-Zahra Hospital in Isfahan, Iran. ICU-acquired candidemia was defined as candidemia with signs and symptoms of infection developing at least 48 h after ICU admission. Candidemia was defined according to previously published definitions. More in detail, candidemia was defined as the presence of at least one positive blood culture for *Candida* species in patients with signs and symptoms of infection [ 16 ]. After that, 8-10 ml of blood samples were taken from each patient under sterile conditions and transferred to blood culture bottles. The bottles were incubated at 35°C, cultured on Sabouraud dextrose agar medium, and monitored on days 1, 2, 3, 7, and 14. The plates were examined for yeast growth. Yeast isolates were transferred to sterile distilled water for molecular identification. A questionnaire was used to collect information about the clinical features and demographic data of the patients. 

The study protocol was approved by the Research Deputy and Ethics Committee of Isfahan University of Medical Sciences, Isfahan, Iran (IR.MUI.REC. 1396.3.603).

### Identification of yeasts

#### Yeast genome extraction

Genomic DNA was extracted and purified using glass bead disruption [ 17 ]. Briefly, the yeast was cultured and suspended in 300 μl of lysis buffer. After adding 300 μl of phenol-chloroform and 200 mg of glass beads (0.5 mm in diameter), the samples were vortexed vigorously for 1 min to disrupt the cells completely. The microtube centrifuged at 10,000 rpm for 5 min, and the aqueous layer was extracted once more with an equal volume of chloroform. Total DNA in the supernatant was precipitated with isopropanol, washed with 70% ethanol, air-dried, and resuspended in 150 μl of TE buffer. This solution was stored at -20°C.

#### PCR-RFLP

PCR-RFLP enzyme method was used to identify the yeasts. To do this, initially, universal primers ITS1 were used with (5`-TCC GTA GGT GAA CCT GCG G-3`) sequence as a forward primer and ITS4 with (5`-TCC TCC GCT TAT TGA TAT GC-3`) sequence as a reverse primer of ITS1-5.8S-ITS2 region of fungal ribosomal region. To prepare the PCR reaction mixture, 10 μl mastermix (Amplicon, Denmark), 1 μl of primers (Sinacolon, Iran) with a concentration of 10 picomol, and 2 μl of extracted DNA were mixed and made up to 25 μl with distilled water. The time and temperature program for the PCR reaction included 5 min at 95°C, 35 cycles for 45 sec at 95°C, 45 sec at 58°C, and 45 sec at 72°C, and at last, a ﬁnal extension step at 72°C for 5 min. 

After the PCR reaction, an RFLP test was performed on the PCR products. Five microliters of PCR products were added to 10 μl of RFLP reaction containing 8.25 μl of distilled water, 1.5 μl of enzyme buffer, and 0.25 μl of *MspI* enzyme (Fermentase, Lithuania) and incubated for 2 h at 37°C. After this step, RFLP products were electrophoresis using 2% agarose gel and TBE buffer for 75 min at 90 volts.

#### Antifungal susceptibility test

Antifungal susceptibility testing was done based on the Clinical and Laboratory Standards Institute Broth Microdilution Guidelines (CLSI-M27-A3 and M60) [ 18 , 19 ]. *C. krusei* (ATCC 6258) and *C. parapsilosis* (ATCC 22019) were used as quality control strains. The categorization of susceptible (S), susceptible-dose dependent (S-DD), intermediate (I), and resistant (R) was derived using breakpoints from the CLSI [ 20 ].

#### Ethics approval and consent to participate

All procedures performed in studies involving human participants were in accordance with the ethical standards of the national research committee and with the 1964 Helsinki Declaration and its later amendments or comparable ethical standards. The study protocol was approved by the Ethics Committee of Isfahan University of Medical Sciences, Isfahan, Iran (IR.MUI.REC.1396.3.603). Informed consent was obtained from all individual participants included in the study.

## Results

Out of 250 patients suspected of candidemia, 22 positive cultures were identified for *Candida* yeast. The age range of patients was from 1 to 93 years, and their mean age was obtained at 50.8 years. The majority of the cases (n=14, 63.6%) were female. The demographic characteristics, types of the underlying disorder, comorbidities with increased risk of candidemia, medications, and outcome of the disease are summarized in Tables 1 and 2. The most common underlying diseases were heart disease and hypertension (36.4%), followed by diabetes (31.8%), dialysis (27.3%), and lung diseases (27.3%). It was found that *C. albicans* was the most isolated species with 12 (54.5%) cases, and the other isolated species were *C. parapsilosis* (n=5, 22.7%) *C. glabrata* (n=4, 18.2%), and *C. tropicalis* (n=1, 4.5%) in descending order. Intravenous catheter was the most common risk factor in patients with candidemia (77.3%), followed by intubation (68.2%) and urinary catheter (40.9%). 

**Table 1:** Underlying diseases and risk factors of candidemia by *Candida* species

	All cases (n=22)	*C. albicans* (n=12)	*C. parapsilosis* (n=5)	*C. glabrata* (n=4)	*C. tropicalis* (n=1)
**Underlying diseases: N (%)**					
Heart diseases	8 (36.4)	5 (41.7)	2 (40)	1 (25)	0
Hypertension	8 (36.4)	4 (33.3)	2 (40)	2 (50)	0
Diabetes	7 (31.8)	3 (25)	2 (40)	2 (50)	0
Pulmonary diseases	6 (27.3)	3 (25)	1 (20)	2 (50)	0
Hemodialysis	6 (27.3)	2 (16.7)	2 (40)	1 (25)	1 (100)
Cancer	4 (18.2)	3 (25)	0	1 (25)	0
**Risk factors**					
Intravenous catheter	17 (77.3)	7 (58.3)	5 (100)	4 (100)	1 (100)
Intubation	15 (68.2)	8 (66.7)	2 (40)	4 (100)	1 (100)
Urinary catheter	9 (40.9)	7 (58.3)	0	2 (50)	0
Neutropenia	3 (13.6)	2 (16.7)	0	1 (25)	0

**Table 2:** Medication and outcome of patients with candidemia in this study

	All cases (n=22)	*C. albicans* (n=12)	*C. parapsilosis* (n=5)	*C. glabrata* (n=4)	*C. tropicalis* (n=1)
**Medication**					
Broad spectrum antibiotic	21 (95.4)	11 (91.7)	5 (100)	4 (100)	1 (100)
Corticosteroids	7 (31.8)	3 (25)	1 (20)	3 (75)	0
Fluconazole	8 (36.4)	4 (33.3)	1 (20)	3 (75)	0
Caspofungin	7 (31.8)	4 (33.3)	1 (20)	2 (50)	0
Amphotericin B	7 (31.8)	3 (25)	2 (40)	2 (50)	0
**Outcome**					
Survived	8 (36.4)	7 (58.3)	1 (20)	0	0
Deceased	14 (63.6)	5 (41.7)	4 (80)	4 (100)	1 (100)

Fever was the most common clinical symptom in patients with candidemia (72.7%). Broad-spectrum antibiotics were prescribed for 95.4% of patients with candidemia. Moreover, in 17 (77.3%) cases, at least one antifungal drug was prescribed. Fluconazole was the most commonly prescribed antifungal. The mortality rate in *Candida* bloodstream infection patients was 63.6%, which was lower when the etiologic agent was *C. albicans*, compared to other species (41.7%). More information on medications and mortality by Candida species were given in Table 2. Based on antimicrobial susceptibility testing of yeasts, *C. albicans* showed high susceptibility to all antifungal agents. In addition, all NAC isolates were susceptible to amphotericin B (Table 3).

**Table 3:** Antifungal susceptibilities of 22 bloodstream clinical isolates of *Candida* species

Species (n)	Antifungal	MIC Range (μg/ml)	% Sensitive
*C. albicans* (12)	Fluconazole	0.125-2	100
	Voriconazole	0.016-0.03	100
	Amphotericin B	0.25-0.5	100
	Caspofungin	0.063-0.125	100
*C. parapsilosis* (5)	Fluconazole	0.25-1.0	100
	Voriconazole	0.016-0.06	100
	Amphotericin B	0.25-0.5	100
	Caspofungin	0.5-4	80
*C. glabrata* (4)	Fluconazole	16-32	0
	Voriconazole	0.125-1	75
	Amphotericin B	0.25-0.5	100
	Caspofungin	0.06-1	75
*C. tropicalis* (1)	Fluconazole	16	0
	Voriconazole	1	0
	Amphotericin B	0.5	100
	Caspofungin	0.125	100

## Discussion

The present study investigated candidemia in patients admitted to the ICU. The prevalence of candidemia in suspected patients was 8.8%. *C. albicans* and *C. parapsilosis* were the most prevalent species isolated from blood samples. *C. albicans* has been repeatedly identified in studies as the most common yeast species [ 7 , 9 , 21 , 22 ]. *C. parapsilosis* was the second Candida species in this study. Mirhandi et al. also reported *C. albicans* and *C. parapsilosis* as more species isolated from patients admitted to the ICU [ 23 ]. 

*C. parapsilosis* is frequently found in the skin of healthy hosts, being the causative agent of catheter-related infections. Moreover, it easily binds to different surfaces (medical equipment and devices), colonize, and form biofilms [ 24 , 25 ]. The increasing prevalence of non-albicans species is a concern because these species have different patterns of drug resistance that make challenges in treatment [ 12 ]. *C. krusei* is inherently resistant to fluconazole and *C. glabrata* is highly resistant to fluconazole [ 26 , 27 ]. 

In this study, all *C. albicans* isolates were susceptible to antifungal agents; however, drug resistance was observed in all NAC species. Furthermore, amphotericin B was the only antifungal effective against all *Candida* isolates. This result has also been obtained in other studies [ 28 - 30 ]. High mortality associated with candidemia can be reduced by prompt and appropriate antifungal therapy. Although in this study, 77.3% of patients had received at least one antifungal drug, the mortality rate was high (63.6%). However, higher mortality rates have also been reported [ 21 ]. Our findings suggest that patients with *C. albicans* candidemia have the lowest mortality rate (41.7%). This finding is consistent with the results of prior studies [ 31 - 33 ]. However, the mortality rate in non-*albicans* species was 90% which could be alarming. Therefore, determining the antifungal susceptibility test and precise identification for yeast isolates is critical for effective treatment. The high mortality in candidemia cases hospitalized in ICU may be due to the severity of the underlying diseases, comorbidities, and poor general condition of patients admitted to the ICUs [ 34 , 35 ]. 

Heart disease and hypertension were the most common underlying diseases in patients with candidemia. Furthermore, intravenous catheter, intubation, and urinary catheter were also the most common risk factors in this study. These risk factors have been reported as the underlying factors in other studies [ 35 , 36 ]. Since most patients in the ICU have an underlying condition, the use of intravascular catheters, intubation, and urinary catheters is common and provides the basis for the colonization of fungal agents. Therefore, these patients must be followed for fungal infections, and prophylaxis with antifungal drugs should be considered. Another risk factor that may play a role in causing candidemia in patients admitted to the ICU is the use of urinary catheters. Urinary catheters have been reported to be involved in 1% to 8% of candidemia cases [ 37 ]. 

The present study has some limitations. First, the number of candidemia cases was limited to investigate the epidemiological patterns of species distribution. Second, antifungal susceptibility patterns with standard methods were not available to address the drug resistance status. Accurate knowledge of predisposing factors and epidemiological patterns can be an effective step in disease management. 

In this study, *C. albicans* is reported to be the most common species causing candidemia; however, an increasing frequency of non-*albicans* species could pose a serious challenge to treatment due to different antifungal susceptibility patterns. Intravascular catheters, urinary catheters, and mechanical ventilation can be considered risk factors for candidiasis in patients admitted to the ICUs. Therefore, following the status of patients admitted to these wards for prevention or timely diagnosis of the disease can be helpful in patient management.

## Conclusion

The incidence of candidemia in ICU patients was high (22 of 250; 9%). Intravascular catheters, urinary catheters, neutropenia, and mechanical ventilation can be considered risk factors for candidiasis in patients admitted to the ICUs. Therefore, following the status of patients admitted to these wards for prevention or timely diagnosis of the disease can be helpful in patient management. With increasing cases of drug resistance in pathogenic yeasts, it is necessary to identify and determine the sensitivity of the drug to these isolates. 

## Acknowledgments

This study was financially supported by the Research Deputy of Isfahan University of Medical Sciences, Isfahan, Iran. The authors are grateful to the staff of Isfahan University of Medical Sciences, Isfahan, Iran.

## Authors’ contribution

A. H. and P. D. contributed to the study design. A. H., P. D., S. A., P.A., and H. R. were responsible for data acquisition. A. H., P. A., and F. A A. evaluated the data and prepared the manuscript. P.D., S.A., H.R., and P.A. conducted the data assessment. All authors read and approved the final manuscript. 

## Conflicts of interest

The authors state that there are no conflicts of interest.

## Financial disclosure

Not applicable.
